# 2516. Development of a de novo Patient-Reported Outcome (PRO) Measure to Assess the Impacts of Disseminated Coccidioidomycosis [Valley Fever] on Patients Living with the Condition

**DOI:** 10.1093/ofid/ofad500.2134

**Published:** 2023-11-27

**Authors:** Emma L Harvey, Mark Bresnik, Tara Symonds, Elliott Blatt, Sophie Hughes, Rob Purdie, Jennifer L Clegg

**Affiliations:** F2G Ltd, Manchester, England, United Kingdom; F2G, Ltd., Princeton, New Jersey; Clinical Outcomes Solutions, Folkestone, England, United Kingdom; Clinical Outcomes Solutions, Folkestone, England, United Kingdom; Clinical Outcomes Solutions, Folkestone, England, United Kingdom; Mycology Advocacy, Research & Education (MYCARE), Bakersfield, California; Clinical Outcomes Solutions, Folkestone, England, United Kingdom

## Abstract

**Background:**

Extrapulmonary or disseminated coccidioidomycosis (cocci) is a rare, severe form of fungal disease acquired by inhaling spores of *Coccidioides* spp., a dimorphic fungus endemic in the southwest United States (US) and parts of Central/South America. Currently, there are no measures that assess the patient experience of health-related quality of life (HRQL) impacts associated with disseminated cocci. Preliminary work to develop a disease-specific PRO has been completed (i.e., literature review, conceptual model development [fig 1], clinician interviews). The focus of this research was to gather qualitative evidence directly from patients living with disseminated cocci.

Initial draft of a Patient-Centered Conceptual Model of Coccidioidomycosis

**Figure:**
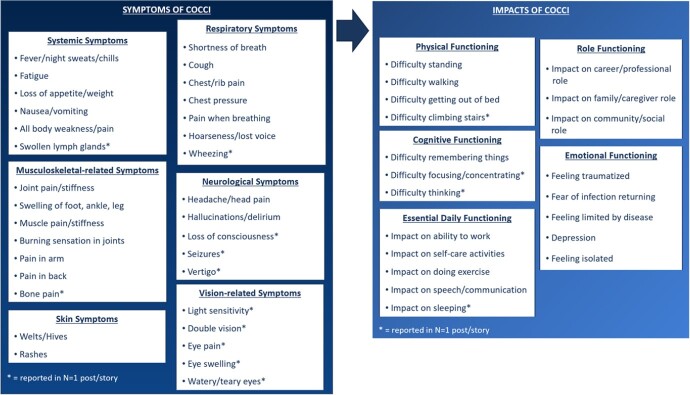

A conceptual model of the symptoms and impacts of coccidioidomycosis was developed by analyzing 29 online patient stories after a literature review revealed qualitative, patient experience research was nonexistent in fungal infections.

**Methods:**

Concept elicitation (CE) interviews were conducted with 25 patients with chronic, symptomatic disseminated cocci. Audio recordings of 60–110-minute telephone interviews were transcribed and qualitatively analyzed using NVivo 1.6. Saturation analysis was performed to determine if all concepts had emerged such that additional interviews would not add to the findings. Item generation meetings were then held between F2G, COS and a patient advocate to develop draft items reflecting the HRQL impacts of the condition.

**Results:**

Thirteen (52.0%) CNS/multi-site and 12 (48.0%) non-CNS/multi-site patients participated; 18 (72.0%) were English-speakers and 7 (28.0%) were native Spanish-speakers; 3 (12.0%) were Black and 12 (48.0%) were Hispanic.

Key impacts were identified across 5 domains, all reaching saturation (fig 2,3): physical function (physical activity, walking, climbing stairs, standing), activities of daily living (household chores, self-care), cognitive function (memory, focus), social-role impacts (work/school, socializing) and emotional (depression, anxiety). No major differences were found between CNS/non-CNS and English/Spanish patient experiences.

Physical function and ADL Impacts Reported by Patients with Disseminated Cocci: Subgroup Analyses 1

**Figure:**
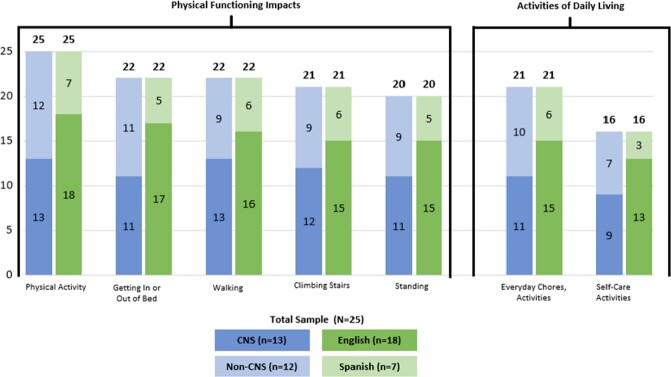

CE interview results showed disseminated patients were significantly impacted in their physical functioning and activities of daily living. A surprising result was that in general, CNS and non-CNS patients had similar impact experiences as a result of disseminated cocci. (Total number of patients who experienced the impact is shown at the top of the column and the number of patients within each subgroup who experienced the impact is shown within the column).

Social/Role, Emotional and Cognitive Impacts Reported by Patients with Disseminated Cocci: Subgroup Analyses 2

**Figure:**
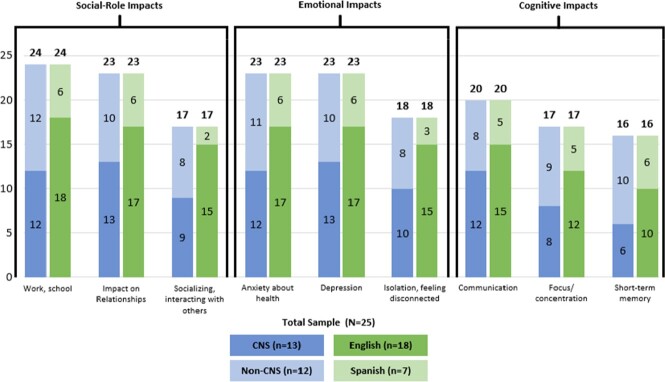

Disseminated patients were also affected socially, cognitively and emotionally, as well as in their roles professionally, and as caregivers and providers. Impact on communication consisted of difficulty retrieving words and following conversation. Cognitive impacts experienced by non-CNS patients were more prevalent than anticipated.

**Conclusion:**

The CE interviews provided rich qualitative data on symptom and impact experiences of patients with disseminated cocci. The findings were used to draft a disease-specific PRO measure assessing HRQL impacts of disseminated cocci, consisting of 19 items across 5 domains (fig 4). Cognitive debrief interviews are now planned with disseminated cocci patients and clinicians to further evaluate the validity of the measure.

Hypothesized Conceptual Framework of PRO measure 'Patient-Reported Impacts of Disseminated Coccidioidomycosis (Valley Fever)'

**Figure:**
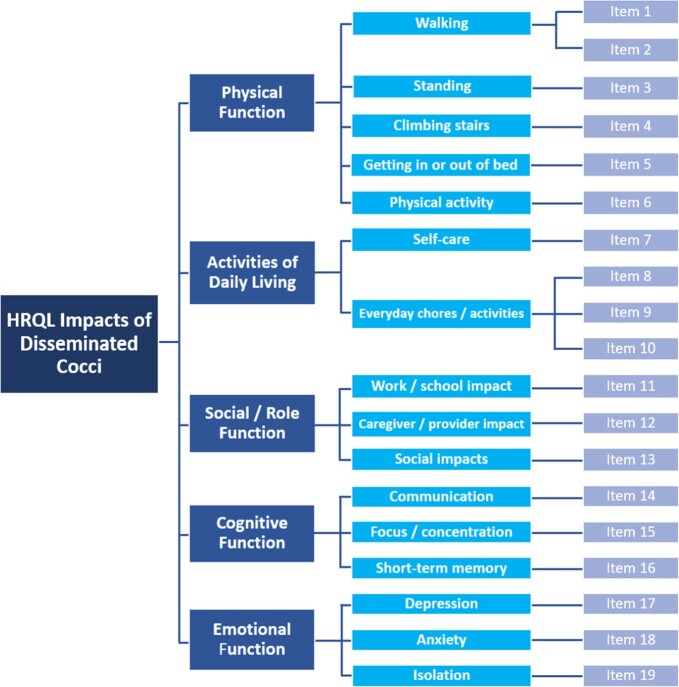

Conceptual frameworks represent how a PRO measure may generate a score, broadly illustrating which patient experiences will be measured and how they will be measured.

**Disclosures:**

**Emma L. Harvey, BSc, MBBS, MRCP, FFPM**, F2G Ltd: Employee|F2G Ltd: Stocks/Bonds **Mark Bresnik, MD**, F2G, Ltd.: Stocks/Bonds **Elliott Blatt, MPH**, F2G Ltd: Advisor/Consultant **Rob Purdie, n/a**, Clinical Outcomes Solutions: Consulting

